# GRASP: A Multitasking Tether

**DOI:** 10.3389/fcell.2016.00001

**Published:** 2016-01-26

**Authors:** Catherine Rabouille, Adam D. Linstedt

**Affiliations:** ^1^Hubrecht Institute, Royal Netherlands Academy of Arts and Sciences (KNAW) and UMC UtrechtUtrecht, Netherlands; ^2^The Department of Cell Biology, University Medical Center UtrechtUtrecht, Netherlands; ^3^Department of Biological Sciences, Carnegie Mellon UniversityPittsburgh, PA, USA

**Keywords:** Golgi organization, PDZ domain, trans-oligomerization, mitosis, unconventional secretion, tether, GRASP, crystal structure

## Abstract

Originally identified as Golgi stacking factors *in vitro*, the Golgi reassembly stacking protein (GRASP) family has been shown to act as membrane tethers with multiple cellular roles. As an update to previous comprehensive reviews of the GRASP family (Giuliani et al., 2011; Vinke et al., 2011; Jarvela and Linstedt, 2012), we outline here the latest findings concerning their diverse roles. New insights into the mechanics of GRASP-mediated tethering come from recent crystal structures. The models of how GRASP65 and GRASP55 tether membranes relate directly to their role in Golgi ribbon formation in mammalian cells and the unlinking of the ribbon at the onset of mitosis. However, it is also clear that GRASPs act outside the Golgi with roles at the ER and ER exit sites (ERES). Furthermore, the proteins of this family display other roles upon cellular stress, especially in mediating unconventional secretion of both transmembrane proteins (Golgi bypass) and cytoplasmic proteins (through secretory autophagosomes).

## Introduction

**Figure 1:**
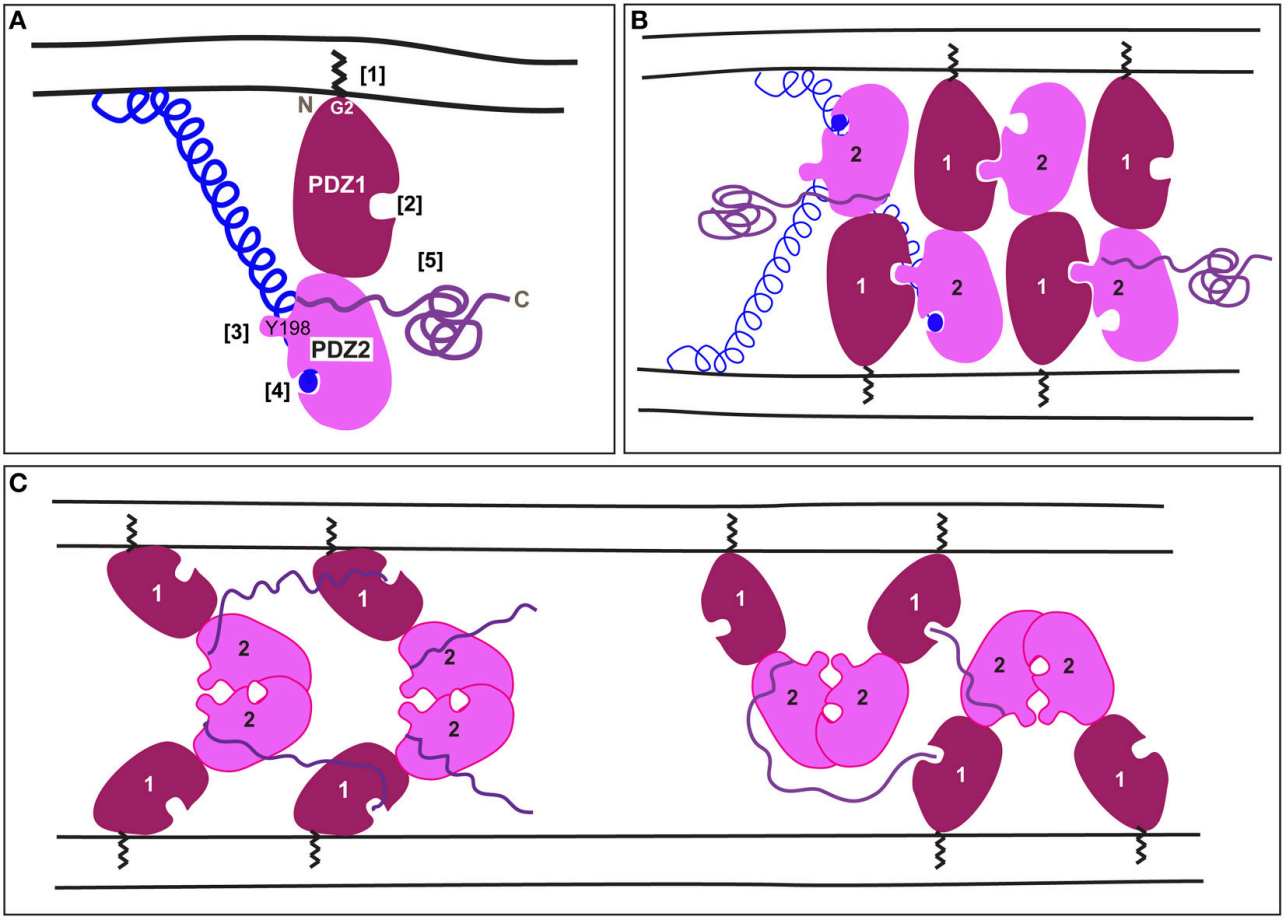
**A schematic representation of mammalian GRASP trans-oligomerization mediating membrane tethering. (A)** A schematic representation of mammalian GRASPs. The zigzag represents the myristate bound to Glycine at position 2 at the N-terminal [1]. PDZ1 (dark red) has a ligand-binding pocket [2] that faces in the opposite direction of the protruding surface around Y198 in PDZ2 (pink) [3]. PDZ2 has also a ligand-binding pocket [4] occupied by the C-terminus of a golgin represented as blue ball and helix (the golgin is GM130 in the case of GRASP65, but less is known about the interaction of golgin45 with GRASP55). The unstructured C-terminal comes out roughly between PDZ1 and PDZ2 [5]. **(B)** According to Truschel et al. (2011), the trans-oligomerization of mammalian GRASPs is mediated by the trans-interaction of the protruding surface of PDZ2 in one GRASP molecule with the PDZ1 binding pocket of another GRASP molecule on the opposite membrane, thus allowing multimerization. Note that the unstructured C-terminal half and the Golgin are not represented for every GRASP molecule. **(C)** According to Feng et al. (2013), GRASP trans-oligomerization is mediated by interaction of two PDZ2 around their binding pockets that face and cover each other. Furthermore, an internal part of the C-terminal tail interacts with the binding pocket of PDZ1. This leads to, at least, two multimerization models. Note that the Golgin interaction has not yet been solved.

Mammalian GRASP65 (GORASP1) and 55 (GORASP2) were first identified as factors required for the *in vitro* stacking of Golgi cisternae (Barr et al., 1997; Shorter et al., 1999) using an assay recapitulating the reassembly of the Golgi at the end of mitosis (Rabouille et al., 1995). They are both about 400 amino-acids long and comprise two functionally separate halves (Barr et al., 1997; Shorter et al., 1999). The N-terminal half contains the largely conserved GRASP domain. The crystal structure of this domain shows that it is comprised of two *bona fide* PDZ (Post synaptic density protein 95, Drosophila disc large tumor suppressor and Zonula occludens-1 protein) domains in tandem (Truschel et al., 2011; Feng et al., 2013; Figure 1A).

On the other hand, the C-terminal half of mammalian GRASP is neither conserved across species nor between themselves, but it does share an enrichment of certain amino-acids including serine, threonine, and proline (Vinke et al., 2011).

Both GRASP55 and GRASP65 strongly localize to the Golgi as peripheral Golgi proteins. Their Golgi recruitment requires both myristoylation of a glycine at position 2 (in all species except yeast and one splice variant of plasmodium) as well as binding to their Golgi receptors that, remarkably, are also peripheral membrane proteins. Importantly, to act in tethering, both GRASP65 and 55 need to be properly oriented on the Golgi membrane, and this is mediated by the two anchoring points, the myristoylation of Glycine2 and the binding to a Golgin (Bachert and Linstedt, 2010; Heinrich et al., 2014). Furthermore, neutron reflection analysis shows that, when properly anchored, the purified GRASP domain stands on end with PDZ1 closest to the membrane (Heinrich et al., 2014; Figure 1A). Removing the myristate allows it to freely rotate in any direction at which point it forms cis interactions and loses the ability to tether.

The golgin Golgin 45 is thought to be the receptor for GRASP55 (Short et al., 2001), whereas the golgin GM130 recruits GRASP65 (Barr et al., 1998; Puthenveedu et al., 2006). The GM130 C-terminus contains a characteristic PDZ-ligand that interacts with binding pocket of PDZ2 in GRASP65 (Bachert and Linstedt, 2010) (Figure 1A). Mutation of PDZ2 of GRASP65 blocks both its GM130 binding and its membrane localization, whereas mutation of PDZ1 does neither. Residues flanking PDZ2 may stabilize the interaction of PDZ2 with GM130 as mutation of these residues also blocks binding (Barr et al., 1998). Surprisingly, a recent co-crystal of the GRASP65 GRASP domain and a GM130 C-terminal peptide has the peptide forming a canonical PDZ interaction with PDZ1 rather than PDZ2 (Hu et al., 2015). Further work is needed to verify the significance of this interaction.

## Transoligomerization and membrane tethering: A crystal structure approach

Mammalian GRASPs function as tethers. By virtue of their trans-dimerization properties, they are able to tether two opposing membrane organelles. Based on *in vitro* and cell-based assays, the consensus was that trans-dimerization occurs when the PDZ1 binding pocket interacts with a conserved sequence of PDZ2 (Sengupta et al., 2009; Sengupta and Linstedt, 2010). Interestingly, the crystal structure of the GRASP55 GRASP domain (at 1.65 Å) shows that this conserved sequence forms a prominent surface projection on PDZ2 comprised of tyrosine (Y198) and a leucine (Truschel et al., 2011). This projection has the correct dimensions to interact with the ligand-binding pocket of PDZ1, leading to multimerization (Figure 1B).

Recently, new crystal structures of the mammalian GRASP domain were reported (GRASP65 at 2.2 Å and GRASP55 at 2.7 Å; Feng et al., 2013) that are very similar to one another and to the original structure (Truschel et al., 2011). However, Feng and colleagues focused on a packing contact evident in their crystals between adjacent PDZ2 domains as, at least, two possible means of GRASP domain oligomerization (Figure 1C). Surprisingly, this contact occludes the PDZ2 binding pocket, which, as noted above, is essential for Golgi localization of GRASP65 and for Golgi ribbon formation. Furthermore, the authors noted that the C-terminal tails of their constructs had inserted into the PDZ1 binding pocket. While this might reflect an additional mode of oligomerization, it is noteworthy that the presence of these sequences at the C-terminus is an artificial consequence of using truncated constructs for crystallization. Given the high protein concentrations reached in crystal trials, the interaction could reflect the promiscuity of PDZ pockets in coordinating free C-termini.

Taken together, more work will be needed to determine the exact basis of mammalian GRASP oligomerization. Indeed, an important caveat is that the structural work to date has only been performed on the isolated GRASP domain. Furthermore, full-length GRASP65 may exist in cells as a dimer (Wang et al., 2003, 2005) and both of the current models can account for multimerization during tethering (Figures 1B,C). As the GRASP domain is well conserved across many multicellular organisms of the animal kingdom, the transoligomerization mechanism is also likely to take place in other species.

## The role of GRASP in mammalian Golgi ribbon formation

The Golgi apparatus in mammalian cells is made of stacks of Golgi cisternae that are linked laterally by small tubules (Képès et al., 2005) forming an interconnected membrane network termed the Golgi ribbon, that is positioned near the nucleus (Klumperman, 2011).

There is still a debate as to whether GRASP65 and 55 act as stacking factors *in vivo*. GRASP knockout or knockdown in non-vertebrate species that only possess a single GRASP does not lead to Golgi unstacking (see Vinke et al., 2011 for refs). For mammalian tissue culture cells, there are conflicting reports regarding the integrity of Golgi stacking after depletion of one or both GRASP proteins (Xiang and Wang, 2010), which is perhaps due to stacking involving multiple factors each of which contributes to the adhesiveness of Golgi membranes (Lee et al., 2014).

**Figure 2:**
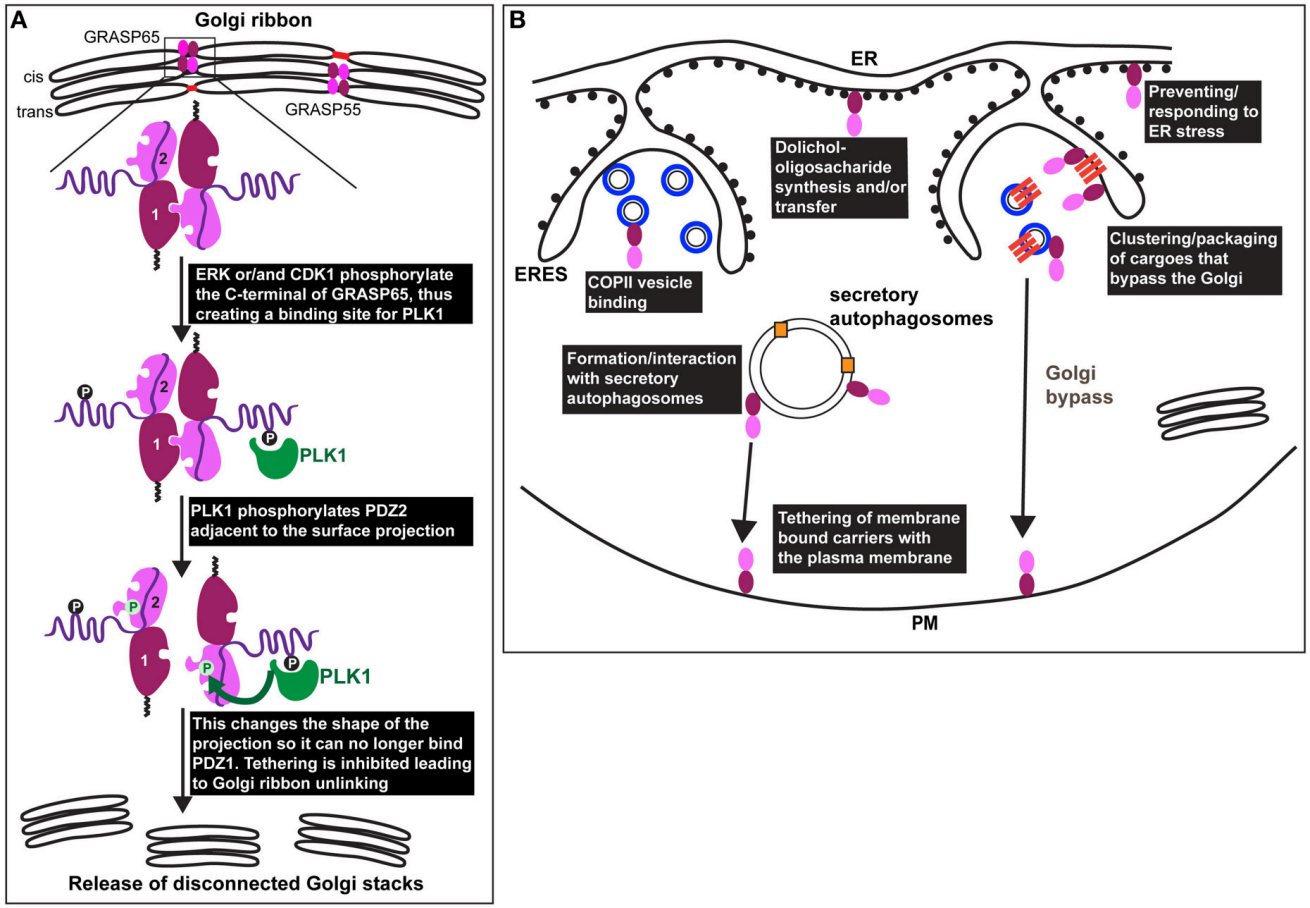
**GRASP in mitosis and under stress. (A)** Representation of the Golgi ribbon as seen in interphase mammalian cells with GRASP65 and 55 (drawn as a dark red/pink bi-lobe as in Figure 1) localized at the cisternal rims (cis for GRASP65 and medial/trans for GRASP55) where they transoligomerize and mediate the formation small tubules (red) laterally connecting adjacent stacks. At the onset of mitosis, GRASP65 and 55 are phosphorylated by ERK and/or CDK1 on residues situated in the C-terminal half. In the case of GRASP65, this creates a binding site for PLK1 (green) that phosphorylates in turn serine 189 near the surface protrusion of PDZ2. This alters the shape of this protrusion blocking its fit in the PDZ1 binding pocket, leading to inhibition of the trans-oligomerization and Golgi ribbon unlinking as observed at G2 *in vivo* (release of disconnected stacks). **(B)** During interphase, GRASPs are also found associated to COPII coated vesicles (drawn as blue circles in ERES), as well as to the ER where they have direct or indirect role in dolichol-oligosaccharide synthesis and/or transfer across the ER membrane. Furthermore, GRASPs are involved in the ER exit of integrin subunits in Drosophila tissues (upon mechanical stress) and of mutant form of CFRT (upon ER stress) in mammals (see text Sorting of Integrins from the ER in Drosophila: A Link to ER Stress and GRASP, and Unconventional Protein Secretion: A Link to Mechanical Stress and Starvation). These transmembrane cargoes are represented as dark orange bars that are clustered in ERES and in COPII coated vesicles. These cargoes are delivered to the plasma membrane without passing through the Golgi (Golgi bypass). GRASPs might mediate the tethering of carriers at the plasma membrane prior their fusion. Last, upon glucose starvation, GRASPs are involved in the formation of, or recruited to, secretory autophagosomes that mediate the unconventional secretion of Acb1 and IL-1ß (pale orange squares) to the extracellular medium. GRASPs might mediate the tethering of the autophagosome at the plasma membrane prior their fusion.

A less contentious role for GRASP65 and 55 is their requirement for Golgi ribbon formation in mammals (Figure 2A). This was first shown in HeLa cells by either depleting GM130 to displace GRASP65 from the Golgi or by depleting GRASP65 itself (Puthenveedu et al., 2006). A similar result was subsequently reported for GRASP55 depletion (Feinstein and Linstedt, 2008). In these depleted cells, the Golgi exists as multiple non-connected, but properly positioned stacks. The defective connectivity was confirmed using FRAP (fluorescence recovery after photobleaching) of GFP tagged cis and trans Golgi enzymes/residents. Whereas, the markers freely move between bleached and non-bleached zones via the tubular connections of an intact Golgi ribbon, their exchange is limited if these connections are not present (Puthenveedu et al., 2006). The dual requirement for GRASP65 and GRASP55 suggests non-redundant functions as might be expected given their localization to distinct parts of the Golgi.

To circumvent possible drawbacks from siRNAs related to specificity and length of time needed for depletion, two approaches were developed. The first is to use the tagging of GRASP65 or 55 with killer-red that allows, upon photo-excitation, the inactivation of the tagged molecule within a couple of minutes. This assay, coupled to the FRAP analysis described above demonstrated that GRASP65 inactivation leads to the Golgi ribbon unlinking specifically at the cis side (Jarvela and Linstedt, 2014), in agreement with its localization (Barr et al., 1997). GRASP55 inactivation, on the other hand, affected the Golgi ribbon more at the trans side (Jarvela and Linstedt, 2014), also in agreement with GRASP55 enrichment at the medial/trans cisternae (Shorter et al., 1999). This supports the non-redundant function of GRASP65 and 55 in the formation/maintenance of the Golgi ribbon.

The second approach was to engineer a protein-null, mutant mouse for GRASP65 (Veenendaal et al., 2014). The homozygous mouse is alive, fertile and healthy, and none of the tissues examined by EM present any alterations in the Golgi ribbon or cisternal stacking. However, when MEFs from these homozygous mutant mice were prepared and functionally tested using the FRAP assay, a defect in Golgi linking specific to cis (but not trans) Golgi cisternae was evidenced (Veenendaal et al., 2014).

## GRASP in mitosis. phosphorylation by PKL1 leads to Golgi ribbon unlinking

One of the arguments strengthening the role of mammalian GRASPs in Golgi ribbon formation is that their phosphorylation by mitotic kinases leads to inhibition of trans dimerization. This results in Golgi ribbon unlinking (severing of the tubules laterally connecting the stacks), not unstacking, a feature identical to GRASP65 and 55 knockdown.

Mammalian GRASPs are major mitotic phosphoproteins and several kinases have been shown to lead to their phosphorylation. For instance, ERK directly phosphorylates GRASP55 (Feinstein and Linstedt, 2007, 2008). Similarly, mutation of ERK phosphorylation sites in GRASP55 to mimic the phosphorylated state blocks GRASP55 activity in both Golgi ribbon formation and trans-dimerization (Feinstein and Linstedt, 2008). GRASP65 is also directly phosphorylated at multiple sites by CDK1, ERK, and PLK1, and phosphorylation blocks its homo-oligomerization *in vitro* (Lin et al., 2000; Sütterlin et al., 2001; Wang et al., 2003; Preisinger et al., 2005; Yoshimura et al., 2005; Tang et al., 2012). This occurs at serine and threonine residues situated in the C-terminal portion of the protein (T216, T237, S241, S274, S291, S373, and S397).

However, the role of CDK1 and ERK mediated phosphorylation in trans-oligomerization has been recently re-assessed using an organelle tethering assay (Sengupta and Linstedt, 2010). In this assay, GRASP65 is targeted to the mitochondrial outer membrane resulting in mitochondria tethering to one another (Sengupta et al., 2009). Critically, the activity depends on the GRASP65 PDZ interaction. Mutating the sites mentioned above to aspartic acid (7XD) in the mito-GRASP65 did not abolish mitochondria tethering, whereas aspartic acid substitution of Ser189 did (Sengupta and Linstedt, 2010). Ser189 is a direct target of PLK1 (Sengupta and Linstedt, 2010) and Ser189 is positioned close to the PDZ ligand centered on Y198. Indeed, the crystal structure of the GRASP domain containing this aspartic acid substitution implies that phosphorylation induces a conformational shift in the surface projection around Y198 such that it no longer fits in the groove of the PDZ1 (Truschel et al., 2012). Since mitotically phosphorylated GRASP65 binds PLK1 (Preisinger et al., 2005), PLK1 may be recruited to GRASP65 by CDK1/ERK phosphorylation so that PLK1 can phosphorylate Ser189 to unlink the Golgi. Consistent with this, HeLa cells expressing a version of GRASP65 that cannot be phosphorylated at Ser189 fail to unlink their Golgi ribbons at the onset of mitosis (Sengupta and Linstedt, 2010; Figure 2A). Accordingly, this prevents/delays cells to enter mitosis (Sütterlin et al., 2002; Duran et al., 2008).

## GRASP under stress

### GRASP at the ER. A role in N-linked glycosylation at the ER

Although GRASPs are considered Golgi proteins, they have been observed at other locations. For instance, the yeast GRASP (Grh1) interacts with COPII subunits in the ERES (Behnia et al., 2007) (Figure 2B). Perhaps related to this, depletion of the mammalian GRASPs in HeLa cells enhances COPII subunit membrane association (Xiang et al., 2013). However, this is also true for the COPI and the clathrin coats, possibly suggesting a pleiotropic effect.

Mammalian GRASPs appear to have a role to play in N-linked glycosylation, not only at the Golgi, but also at the initial steps of the process in the ER. Through a thorough analysis of the N-glycans by mass spectrometry, depletion of GRASP55 (but not GRASP65) was shown to result in a substantial decrease in N-linked glycans borne by glycoproteins. This effect was even more pronounced when both GRASP55 and 65 were depleted (Xiang et al., 2013). There was a reduction in both complex oligosaccharides and high mannose (ER) forms suggesting that upon GRASP55/65 depletion, co-translational initiation of glycoprotein glycosylation in the ER is compromised (Xiang et al., 2013). These glycosylation defects are thought to be due to a lack of the N-glycan donor dolichol, either because GRASP55 affects the synthesis of the donor dolichol or its transfer from the cytoplasm to the ER lumen (Figure 2B). Defects in glycosylation result in protein misfolding and ER stress (see below Wang et al., 2015).

### Sorting of integrins from the ER in drosophila: A link to ER stress

GRASP family members have been shown to play a role in the sorting of certain transmembrane proteins from the ER. Indeed, in a Drosophila mutant for dGRASP (that encodes the single Drosophila homolog of mammalian GRASP65 and 55), the integrin subunit αPS1 that is normally delivered to the plasma membrane, was specifically retained in the ER of the follicle cells covering the oocyte in Drosophila ovaries (Schotman et al., 2008). Strikingly, the same phenotype has been recently observed in larval muscle fibers for integrin αPS2 (Wang et al., 2015), suggesting a role for dGRASP in the sorting/packaging of integrin subunits from the ER to downstream compartments in certain Drosophila tissues (Figure 2B).

In the muscle fibers, the role of dGRASP in eliciting the exit of αPS2 from the ER at ERES was further elucidated (Wang et al., 2015). Interestingly, dGRASP interacts with Clueless. *clueless* mutants have the same phenotype as this of dGRASP loss of function. Although Clueless normally function in mitochondria (Cox and Spradling, 2009), Clueless function in integrin sorting is distinct from it and dGRASP is not involved in the mitochondria phenotype (Wang et al., 2015).

There are three possibilities to explain the ER retention of integrins in the ER in the absence of dGRASP: (1) GRASP65/55 interact with P24 oligomers (Barr et al., 2001) that conceivably serve as cargo receptors for integrins at the ER and ERES (Kuiper et al., 2001). (2) The ER retention could be due to glycosylation defects if absence of dGRASP impairs glycosylation (as it does in mammalian cells, see above). (3) Similar to the situation in muscle, loss of dGRASP (and Clu) may trigger ER stress and Sec16 degradation thereby compromising COPII vesicle assembly (Sprangers and Rabouille, 2015) and integrin export out of the ER. Indeed, ER stress could affect many ER functions that contribute to impaired sorting of integrin subunits.

### GRASP and unconventional protein secretion: A link to mechanical stress and starvation

Interestingly, it is becoming increasingly clear that in addition to the classical ER>Golgi>plasma membrane secretory pathway, proteins can be delivered to the plasma membrane or the extracellular space in an unconventional manner (Rabouille et al., 2012). Strikingly, out of the four mechanisms proposed, GRASPs are involved in two that are elicited by cellular stress: the Golgi bypass of transmembrane proteins and the secretion of leaderless peptide proteins to the extracellular medium by autophagosome-like structures (Rabouille et al., 2012).

#### GRASP and Golgi bypass of transmembrane proteins

Although αPS1 is normally delivered to the plasma membrane via the classical secretory pathway, it is unconventionally secreted at specific stages of Drosophila follicle epithelium development where it becomes both insensitive to BFA and to the loss of Syntaxin5 (Schotman et al., 2008), suggesting that it bypasses the Golgi (Grieve and Rabouille, 2014; Figure 2B). At the same stages, dGRASP is needed for αPS1 exit from the ER, suggesting that dGRASP somehow mediates its packaging into vesicles and/or the tethering of these vesicles to specific plasma membrane domains (Figure 2B). This is supported by the finding that dGRASP mRNA is enriched near these plasma membrane domains, likely leading to its local synthesis (Schotman et al., 2008). Interestingly, the targeted localization of dGRASP mRNA near the plasma membrane is triggered by mechanical stress (Schotman et al., 2009; Giuliani et al., 2014), suggesting that this might be the trigger for the integrin subunit Golgi bypass.

GRASP55 (and 65) have also been shown to mediate the Golgi bypass of another transmembrane protein delivered to the plasma membrane, the ΔF508 mutated form of CFTR (cystic fibrosis transmembrane conductance regulator) in mammalian cells (Gee et al., 2011). Normally, this mutated protein is retained in the ER, but under conditions affecting Golgi function (Syntaxin 5 depletion, Brefeldin A treatment) or exit from the ER (expression of dominant negative Sar1), this mutant protein bypasses the Golgi to reach the plasma membrane. All these conditions cause ER stress and, remarkably, it is the specific triggering of ER stress that causes mutant CFTR to reach the plasma membrane through a Golgi bypass route. Strikingly, this trafficking requires GRASP55, and overexpression of GRASP55 in CFTR mouse model rescues viability (Gee et al., 2011). In the absence of GRASP55, mutant CFTR is stuck in the ER, similar to the situation in Drosophila.

However, these data are controversial as the form of GRASP55 used to perform these experiments was tagged at the N-terminus, which is incompatible with myristoylation of G2 (see above) and prevents GRASP55 anchoring to the Golgi membrane (Barr et al., 1997; Shorter et al., 1999; Kondylis et al., 2005; Heinrich et al., 2014). Whether N-terminal tagging interferes with GRASP55 function at the ER remains unclear. Furthermore, the conclusion that GRASP binds CFTR via a PDZ interaction is questionable because the binding experiments used GRASP constructs that were mistakenly truncated such that the key ß2 strands forming the PDZ binding pockets were excluded.

#### GRASP and unconventional secretion of cytoplasmic proteins by secretory autophagosomes

In Dictyostelium, AcbA (acetyl CoA binding protein A) is required to be released in the extracellular medium for stalk formation upon starvation conditions, but this release is inhibited in a GRASP (GrpA) mutant (Kinseth et al., 2007). Similarly, Acb1 release from starved yeast was also inhibited in Grh1 mutant (Duran et al., 2010; Manjithaya et al., 2010). As AcbA/Acb1 are leaderless peptide small proteins, these data suggest that GRASP family members, at least in these two species, are required for the unconventional secretion of cytoplasmic proteins, independently of the classical secretory pathway.

Using yeast genetics, the pathway has been dissected and involves the formation of starvation dependent specialized autophagosomes called CUPS (compartment for unconventional protein secretion) near ER exit sites (ERES). CUPS are enriched in autophagosomal markers and they require both autophagy and ESCRT machinery for their formation (Bruns et al., 2011). Grh1 is also required for CUPS formation where it is enriched and where it could act to tether these structures to the plasma membrane (Duran et al., 2010; Figure 2B). Indeed, unlike classical autophagosomes, which fuse with endosomes/lysosomes, CUPS are thought to fuse with the plasma membrane to allow delivery of their content to the extracellular medium.

Interestingly, a role for GRASP has also been shown for the release of IL-1ß in mammalian cells via secretory autophagosomes. IL-1ß is known to be unconventionally secreted and one of the proposed mechanisms has been its sequestration in endosomes by micro- or macro- autophagy (Nickel and Rabouille, 2009). Therefore, the dual requirement for GRASP55 and Atg5, a gene essential for autophagy, strengthen the yeast findings (Dupont et al., 2011).

However, the secretion of IL-1ß is not simply based on the engulfment of parts of the cytoplasm. It appears to require the specific translocation of IL1ß between the two membranes of early autophagosomes. This translocation relies on the motif KFERQ, and concentrates and protects the protein before its release in the extracellular medium (Zhang et al., 2015). Whether Acb1/A contains such a motif will need to be determined. The role of GRASP has further been confirmed in the secretion of mature IL-1ß (Zhang et al., 2015). But whether it plays a role in the tethering of the secretory autophagosomes prior their fusion with the plasma membrane or in the capture of the cargo is still not addressed.

## GRASP: A tether under stress?

Given the clear evidence for numerous roles carried out by the GRASP family members in different species, especially under cellular stress, the question arises as to whether the GRASPs act as membrane tethers in all of these processes. Furthermore, if GRASPs do tether when functioning outside the Golgi, are the tethering complexes homomeric (akin to the models in Figure 1) or are they comprised of an entirely new set of interacting partners? The latter is a distinct possibility given the potential of the PDZ domains to bind diverse ligands as well as the disordered tail domain having the flexibility to interact with many partners. Thus, an important future direction is to elucidate the detailed molecular mechanism underlying the many GRASP-dependent functions.

## Author contributions

All authors listed, have made substantial, direct and intellectual contribution to the work, and approved it for publication.

## Funding

Work in the Rabouille's lab is funded by Dutch Organization of Scientific Research (NWO) (912080241). Work in the Linstedt lab is funded by NIH R01 grants GM095549 and GM056779.

### Conflict of interest statement

The authors declare that the research was conducted in the absence of any commercial or financial relationships that could be construed as a potential conflict of interest.

## References

[B1] Bachert C. Linstedt A. D. (2010). Dual anchoring of the GRASP membrane tether promotes trans pairing. J. Biol. Chem. 285, 16294–16301. 10.1074/jbc.M110.116129 20228057 PMC2871497

[B2] Barr F. A. Nakamura N. Warren G. (1998). Mapping the interaction between GRASP65 and GM130, components of a protein complex involved in the stacking of Golgi cisternae. EMBO J. 17, 3258–3268. 10.1093/emboj/17.12.3258 9628863 PMC1170664

[B3] Barr F. A. Preisinger C. Kopajtich R. Körner R. (2001). Golgi matrix proteins interact with p24 cargo receptors and aid their efficient retention in the Golgi apparatus. J. Cell Biol. 155, 885–891. 10.1083/jcb.200108102 11739402 PMC2150891

[B4] Barr F. A. Puype M. Vandekerckhove J. Warren G. (1997). GRASP65, a protein involved in the stacking of Golgi cisternae. Cell 91, 253–262. 10.1016/S0092-8674(00)80407-9 9346242

[B5] Behnia R. Barr F. A. Flanagan J. J. Barlowe C. Munro S. (2007). The yeast orthologue of GRASP65 forms a complex with a coiled-coil protein that contributes to ER to Golgi traffic. J. Cell Biol. 176, 255–261. 10.1083/jcb.200607151 17261844 PMC2063951

[B6] Bruns C. McCaffery J. M. Curwin A. J. Duran J. M. Malhotra V. (2011). Biogenesis of a novel compartment for autophagosome-mediated unconventional protein secretion. J. Cell Biol. 195, 979–992. 10.1083/jcb.201106098 22144692 PMC3241719

[B7] Cox R. T. Spradling A. C. (2009). Clueless, a conserved Drosophila gene required for mitochondrial subcellular localization, interacts genetically with parkin. Dis. Model. Mech. 2, 490–499. 10.1242/dmm.002378 19638420 PMC2737057

[B8] Dupont N. Jiang S. Pilli M. Ornatowski W. Bhattacharya D. Deretic V. (2011). Autophagy-based unconventional secretory pathway for extracellular delivery of IL-1beta. EMBO J. 30, 4701–4711. 10.1038/emboj.2011.398 22068051 PMC3243609

[B9] Duran J. M. Anjard C. Stefan C. Loomis W. F. Malhotra V. (2010). Unconventional secretion of Acb1 is mediated by autophagosomes. J. Cell Biol. 188, 527–536. 10.1083/jcb.200911154 20156967 PMC2828925

[B10] Duran J. M. Kinseth M. Bossard C. Rose D. W. Polishchuk R. Wu C. C.. (2008). The role of GRASP55 in Golgi fragmentation and entry of cells into mitosis. Mol. Biol. Cell. 19, 2579–2587. 10.1091/mbc.E07-10-0998 18385516 PMC2397314

[B11] Feinstein T. N. Linstedt A. D. (2007). Mitogen-activated protein kinase kinase 1-dependent Golgi unlinking occurs in G2 phase and promotes the G2/M cell cycle transition. Mol. Biol. Cell 18, 594–604. 10.1091/mbc.E06-06-0530 17182854 PMC1783781

[B12] Feinstein T. N. Linstedt A. D. (2008). GRASP55 regulates Golgi ribbon formation. Mol. Biol. Cell 19, 2696–2707. 10.1091/mbc.E07-11-1200 18434598 PMC2441664

[B13] Feng Y. Yu W. Li X. Lin S. Zhou Y. Hu J.. (2013). Structural insight into Golgi membrane stacking by GRASP65 and GRASP55 proteins. J. Biol. Chem. 288, 28418–28427. 10.1074/jbc.M113.478024 23940043 PMC3784759

[B14] Gee H. Y. Noh S. H. Tang B. L. Kim K. H. Lee M. G. (2011). Rescue of DeltaF508-CFTR trafficking via a GRASP-dependent unconventional secretion pathway. Cell 146, 746–760. 10.1016/j.cell.2011.07.021 21884936

[B15] Giuliani F. Grieve A. Rabouille C. (2011). Unconventional secretion: a stress on GRASP. Curr. Opin. Cell Biol. 23, 498–504. 10.1016/j.ceb.2011.04.005 21571519

[B16] Giuliani G. Giuliani F. Volk T. Rabouille C. (2014). The Drosophila RNA-binding protein HOW controls the stability of dgrasp mRNA in the follicular epithelium. Nucleic Acids Res. 42, 1970–1986. 10.1093/nar/gkt1118 24217913 PMC3919595

[B17] Grieve A. G. Rabouille C. (2014). Extracellular cleavage of E-cadherin promotes epithelial cell extrusion. J. Cell Sci. 127, 3331–3346. 10.1242/jcs.147926 24895403

[B18] Heinrich F. Nanda H. Goh H. Z. Bachert C. Lösche M. Linstedt A. D. (2014). Myristoylation restricts orientation of the GRASP domain on membranes and promotes membrane tethering. J. Biol. Chem. 289, 9683–9691. 10.1074/jbc.M113.543561 24505136 PMC3975017

[B19] Hu F. Shi X. Li B. Huang X. Morelli X. Shi N. (2015). Structural basis for the interaction between the Golgi Reassembly-Stacking Protein GRASP65 and the Golgi Matrix Protein GM130. J. Biol. Chem. 290, 26373–26382. 10.1074/jbc.M115.657940 26363069 PMC4646294

[B20] Jarvela T. Linstedt A. D. (2012). Golgi GRASPs: moonlighting membrane tethers. Cell Health Cytoskelet. 4, 37–47. 10.2147/CHC.S21849

[B21] Jarvela T. Linstedt A. D. (2014). Isoform-specific tethering links the Golgi ribbon to maintain compartmentalization. Mol. Biol. Cell 25, 133–144. 10.1091/mbc.E13-07-0395 24227884 PMC3873884

[B22] Képès F. Rambourg A. Satiat-Jeunemaître B. (2005). Morphodynamics of the secretory pathway. Int. Rev. Cytol. 242, 55–120. 10.1016/S0074-7696(04)42002-6 15598467

[B23] Kinseth M. A. Anjard C. Fuller D. Guizzunti G. Loomis W. F. Malhotra V. (2007). The Golgi-associated protein GRASP is required for unconventional protein secretion during development. Cell 130, 524–534. 10.1016/j.cell.2007.06.029 17655921

[B24] Klumperman J. (2011). Architecture of the mammalian Golgi. Cold Spring Harb. Perspect. Biol. 3:a005181. 10.1101/cshperspect.a005181 21502307 PMC3119909

[B25] Kondylis V. Spoorendonk K. M. Rabouille C. (2005). dGRASP localization and function in the early exocytic pathway in Drosophila S2 cells. Mol. Biol. Cell 16, 19870–19875. 10.1091/mbc.E04-10-0938 15975913 PMC1196319

[B26] Kuiper R. P. Bouw G. Janssen K. P. Rötter J. van Herp F. Martens G. J. (2001). Localization of p24 putative cargo receptors in the early secretory pathway depends on the biosynthetic activity of the cell. Biochem. J. 360, 421–429. 10.1042/bj3600421 11716771 PMC1222243

[B27] Lee I. Tiwari N. Dunlop M. H. Graham M. Liu X. Rothman J. E. (2014). Membrane adhesion dictates Golgi stacking and cisternal morphology. Proc. Natl. Acad. Sci. U.S.A. 111, 1849–1854. 10.1073/pnas.1323895111 24449908 PMC3918774

[B28] Lin C. Y. Madsen M. L. Yarm F. R. Jang Y. J. Liu X. Erikson R. L. (2000). Peripheral Golgi protein GRASP65 is a target of mitotic polo-like kinase (Plk) and Cdc2. Proc. Natl. Acad. Sci. U.S.A. 97, 12589–12594. 10.1073/pnas.220423497 11050165 PMC18808

[B29] Manjithaya R. Anjard C. Loomis W. F. Subramani S. (2010). Unconventional secretion of *Pichia pastoris* Acb1 is dependent on GRASP protein, peroxisomal functions, and autophagosome formation. J. Cell Biol. 188, 537–546. 10.1083/jcb.200911149 20156962 PMC2828923

[B30] Nickel W. Rabouille C. (2009). Mechanisms of regulated unconventional protein secretion. Nat. Rev. Mol. Cell Biol. 10, 148–155. 10.1038/nrm2617 19122676

[B31] Preisinger C. Körner R. Wind M. Lehmann W. D. Kopajtich R. Barr F. A. (2005). Plk1 docking to GRASP65 phosphorylated by Cdk1 suggests a mechanism for Golgi checkpoint signalling. EMBO J. 24, 753–765. 10.1038/sj.emboj.7600569 15678101 PMC549625

[B32] Puthenveedu M. A. Bachert C. Puri S. Lanni F. Linstedt A. D. (2006). GM130 and GRASP65-dependent lateral cisternal fusion allows uniform Golgi-enzyme distribution. Nat. Cell Biol. 8, 238–248. 10.1038/ncb1366 16489344

[B33] Rabouille C. Malhotra V. Nickel W. (2012). Diversity in unconventional protein secretion. J. Cell Sci. 125, 5251–5255. 10.1242/jcs.103630 23377655

[B34] Rabouille C. Misteli T. Watson R. Warren G. (1995). Reassembly of Golgi stacks from mitotic Golgi fragments in a cell-free system. J. Cell Biol. 129, 605–618. 10.1083/jcb.129.3.605 7730399 PMC2120448

[B35] Schotman H. Karhinen L. Rabouille C. (2008). dGRASP-mediated noncanonical integrin secretion is required for Drosophila epithelial remodeling. Dev. Cell 14, 171–182. 10.1016/j.devcel.2007.12.006 18267086

[B36] Schotman H. Karhinen L. Rabouille C. (2009). Integrins mediate their unconventional, mechanical-stress-induced secretion via RhoA and PINCH in Drosophila. J. Cell Sci. 122, 2662–2672. 10.1242/jcs.039347 19584096

[B37] Sengupta D. Linstedt A. D. (2010). Mitotic inhibition of GRASP65 organelle tethering involves Polo-like kinase 1 (PLK1) phosphorylation proximate to an internal PDZ ligand. J. Biol. Chem. 285, 39994–40003. 10.1074/jbc.M110.189449 20937827 PMC3000981

[B38] Sengupta D. Truschel S. Bachert C. Linstedt A. D. (2009). Organelle tethering by a homotypic PDZ interaction underlies formation of the Golgi membrane network. J. Cell Biol. 186, 41–55. 10.1083/jcb.200902110 19581411 PMC2712994

[B39] Short B. Preisinger C. Körner R. Kopajtich R. Byron O. Barr F. A. (2001). A GRASP55-rab2 effector complex linking Golgi structure to membrane traffic. J. Cell Biol. 155, 877–883. 10.1083/jcb.200108079 11739401 PMC2150909

[B40] Shorter J. Watson R. Giannakou M. E. Clarke M. Warren G. Barr F. A. (1999). GRASP55, a second mammalian GRASP protein involved in the stacking of Golgi cisternae in a cell-free system. J. Cell Biol. 18, 4949–4960. 10.1093/emboj/18.18.4949 10487747 PMC1171566

[B41] Sprangers J. Rabouille C. (2015). SEC16 in COPII coat dynamics at ER exit sites. Biochem. Soc. Trans. (UK). 43, 97–103. 10.1042/BST20140283 25619252

[B42] Sütterlin C. Lin C. Y. Feng Y. Ferris D. K. Erikson R. L. Malhotra V. (2001). Polo-like kinase is required for the fragmentation of pericentriolar Golgi stacks during mitosis. Proc. Natl. Acad. Sci. U.S.A. 98, 9128–9132. 10.1073/pnas.161283998 11447294 PMC55384

[B43] Sütterlin C. Hsu P. Mallabiabarrena A. Malhotra V. (2002). Fragmentation and dispersal of the pericentriolar Golgi complex is required for entry into mitosis in mammalian cells. Cell 109, 359–369. 10.1016/S0092-8674(02)00720-1 12015985

[B44] Tang D. Yuan H. Vielemeyer O. Perez F. Wang Y. (2012). Sequential phosphorylation of GRASP65 during mitotic Golgi disassembly. Biol. Open 1, 1204–1214. 10.1242/bio.20122659 23259055 PMC3522882

[B45] Truschel S. T. Sengupta D. Foote A. Heroux A. Macbeth M. R. Linstedt A. D. (2011). Structure of the membrane-tethering GRASP domain reveals a unique PDZ ligand interaction that mediates Golgi biogenesis. J. Biol. Chem. 286, 20125–20129. 10.1074/jbc.C111.245324 21515684 PMC3121478

[B46] Truschel S. T. Zhang M. Bachert C. Macbeth M. R. Linstedt A. D. (2012). Allosteric regulation of GRASP protein-dependent Golgi membrane tethering by mitotic phosphorylation. J. Biol. Chem. 287, 19870–19875. 10.1074/jbc.M111.326256 22523075 PMC3370171

[B47] Veenendaal T. Jarvela T. Grieve A. G. van Es J. H. Linstedt A. D. Rabouille C. (2014). GRASP65 controls the cis Golgi integrity *in vivo*. Biol. Open 3, 431–443. 10.1242/bio.20147757 24795147 PMC4058077

[B48] Vinke F. P. Grieve A. G. Rabouille C. (2011). The multiple facets of the Golgi reassembly stacking proteins. Biochem. J. 434, 423–433. 10.1042/BJ20101540 21235525

[B49] Wang Y. Satoh A. Warren G. (2005). Mapping the functional domains of the Golgi stacking factor GRASP65. J. Biol. Chem. 280, 4921–4928. 10.1074/jbc.M412407200 15576368 PMC4443495

[B50] Wang Y. Seemann J. Pypaert M. Shorter J. Warren G. (2003). A direct role for GRASP65 as a mitotically regulated Golgi stacking factor. EMBO J. 22, 3279–3290. 10.1093/emboj/cdg317 12839990 PMC165642

[B51] Wang Z. H. Rabouille C. Geisbrecht E. R. (2015). Loss of a Clueless-dGRASP complex results in ER stress and blocks Integrin exit from the perinuclear endoplasmic reticulum in Drosophila larval muscle. Biol. Open 4, 636–648. 10.1242/bio.201511551 25862246 PMC4434815

[B52] Xiang Y. Wang Y. (2010). GRASP55 and GRASP65 play complementary and essential roles in Golgi cisternal stacking. J. Cell Biol. 188, 237–251. 10.1083/jcb.200907132 20083603 PMC2812519

[B53] Xiang Y. Zhang X. Nix D. B. Katoh T. Aoki K. Tiemeyer M.. (2013). Regulation of protein glycosylation and sorting by the Golgi matrix proteins GRASP55/65. Nat. Commun. 4:1659. 10.1038/ncomms2669 23552074 PMC3620728

[B54] Yoshimura S. Yoshioka K. Barr F. A. Lowe M. Nakayama K. Ohkuma S.. (2005). Convergence of cell cycle regulation and growth factor signals on GRASP65. J. Biol. Chem. 280, 23048–23056. 10.1074/jbc.M502442200 15834132

[B55] Zhang M. Kenny S. Ge L. Xu K. Schekman R. (2015). Translocation of interleukin-1beta into a vesicle intermediate in autophagy-mediated secretion. Elife 4:e11205. 10.7554/eLife.11205 26523392 PMC4728131

